# Microbial Changes Occurring During Oronasal Fistula Wound Healing

**DOI:** 10.3390/microorganisms13020327

**Published:** 2025-02-02

**Authors:** Steven L. Goudy, Heath L. Bradley, Camilo Anthony Gacasan, Afra Toma, Keerthi Priya Chinniampalayam Sekar, William M. Wuest, Martin Tomov, Vahid Serpooshan, Ahmet Coskun, Rheinallt M. Jones

**Affiliations:** 1 Department of Otolaryngology, Emory University, Children’s Healthcare of Atlanta, Atlanta, GA 30329, USA; 2 Department of Pediatrics, Emory University, Children’s Healthcare of Atlanta, Atlanta, GA 30329, USA; 3 Department of Biomedical Engineering, Emory University and Georgia Institute of Technology, Atlanta, GA 30332, USA; 4 Department of Chemistry, Emory University, Atlanta, GA 30329, USA; william.wuest@emory.edu

**Keywords:** oro-nasal fistula (ONF), oral microbiome, antibiotics

## Abstract

The oral microbiome is a complex community that matures with dental development and is recognized as a risk factor for systemic disease. Despite the oral cavity having a substantial microbial burden, healing of superficial oral wounds occurs quickly and with little scarring. By contrast, creation of an oro-nasal fistula (ONF), often occurring after surgery to correct a cleft palate, is a significant wound healing challenge. Methods: In this study, we characterized the changes in the oral microbiome of mice following a freshly inflicted wound in the oral palate that results in an open and unhealed ONF. Results: Creation of an ONF in mice significantly lowered oral microbiome alpha diversity, with concurrent blooms of *Enterococcus faecalis*, *Staphylococcus lentus*, and Staphylococcus xylosus in the oral cavity. Treatment with oral antibiotics one week before ONF infliction reduced microbiome alpha diversity and prevented *E. faecalis*, *S. lentus*, and S. xylosus blooms, but did not impact ONF healing. Conclusions: An ONF in the murine palate leads to a dysbiotic oral microbiome and a bloom of opportunistic pathogens that may prevent ONF healing. Delivery of therapeutics that accelerate ONF healing might restore oral microbiome diversity and inhibit blooms of opportunistic pathogens.

## 1. Introduction

The oral cavity has the second most diverse microbiota after the gut, with the community structure totaling over 700 species of bacteria. The oral microbiome is crucial in maintaining oral, as well as systemic, health. Initial colonizers immediately after birth include mainly aerobes such as *Streptococcus*, Lactobacillus, Actinomyces, Neisseria and *Veillonella*. Thereafter, following tooth eruption with plaque accumulation and the development of gingival crevices, wider species diversity and microbial succession occurs within the oral cavity until microbes from the phyla *Firmicutes*, *Proteobacteria*, *Bacteroidetes*, *Actinobacteria*, *Fusobacteria*, and *Neisseria* predominately inhabit the oral cavity [1,2,3,4]. However, less is known about the oral microbiome in relation to large oral mucosal injury as would occur following cleft palate corrective surgery, although a recent report on the oral microbiome of children with cleft lip and palate revealed associations of certain microbial taxa with the extent of wound inflammation [5]. After oral surgeries such as cleft palate repair, antibiotics are often given prophylactically, but it remains unknown whether antibiotics impact wound healing or microbiome composition after the procedure. Pre-operative assessment of oral surgery patients does not routinely perform surveillance on the oral microbial composition or mitigation of associated periodontal disease despite the association of microbiome dysbiosis with poor wound healing [6].

Adverse wound healing after cleft palate repair occurs in up to 60% of children, leading to the formation of an oro-nasal fistula (ONF). An ONF is a direct opening between the mouth and the nose that requires multiple follow-up surgeries to repair. Heterogeneity in oral wound healing outcomes following oral surgery is poorly understood, although factors that are associated with the occurrence of an oro-nasal fistula (ONF) are known to function in healing of the rotated mucosal flaps, including wound tension, width of the cleft palate, and nutritional status [7]. In addition, variations in patient-specific composition of the oral microbiome and colonization by oral pathogens have all been implicated in ONF formation. In this study, our goal is to employ a mouse model of oral wound injury to characterize the major changes to the composition of the oral microbiome following the infliction of an ONF to the oral palate. We will also assess the impact of pre-treatment of mice with broad-spectrum antibiotics on ONF healing.

## 2. Materials and Methods

Experimental Mice. All experiments were conducted using C57BL/6 mice purchased from The Jackson Laboratories. All procedures were approved by The Emory University Institutional Care and Use Committee (IACUC), which provides oversight over the humane and ethical animal care and use of animals in research (Protocol #201700263). All data are reported as recommended by the ARRIVE guidelines, including clear identification of control groups, exact sample size number, and inclusion and exclusion criteria where relevant. Mice were randomly assigned to experimental groups, the investigators analyzing mouse samples were blind to the treatment group, and outcome measures for all experiments are outlined. Mice were housed in a controlled, specialized vivarium designed for laboratory animals, with precise monitoring of temperature, humidity, and lighting to ensure the health and well-being of the mice. Mice were monitored daily by both investigative and veterinary staffs to confirm the well-being of mice used in the outlined experiments. Where specified, mice were treated with and antibiotic cocktail of 1 mg/mL Ampicillin, 0.5 mg/mL Vancomycin, 1 mg/mL Neomycin, and 1 mg/mL Metronidazole included in their ad libitum drinking water.

Mouse ONF Model. Twelve-week-old anesthetized C57BL/6 mice were placed in a supine position, and a dissecting microscope was placed to visualize the oral cavity. An eyelid retractor was used to retract the upper and lower jaw with the tongue pushed out of the way. Using an ophthalmologic cautery, a 1.5 mm oronasal fistula was performed between the first molars in the midline and hemostasis was achieved. Healing rates of the 1.5 mm oronasal fistula were followed daily for up to 7 days before sacrifice.

Analysis of Microbiome sequencing 16S rDNA sequencing. Oral microbiome samples were collected from 12-week-old mice using swabs. The swabs were analyzed through sequencing of the 16S rDNA gene undertaken by ZymoBIOMICS (Zymo Research, Irvine, CA, USA). DNA was extracted from swabs using ZymoBIOMICS-96 MagBead DNA kit (Zymo Research, Irvine, CA, USA). The ZymoBIOMICS Microbial Community Standard was used as a positive control for each DNA extraction. The ZymoBIOMICS Microbial Community DNA Standard was used as a positive control for each targeted library preparation. Negative controls were included to assess the level of bioburden carried by the wet-lab process. The DNA samples were then prepared for targeted sequencing with Quick-16S Primer Set V3-V4 Library (Zymo Research, Irvine, CA, USA). The sequencing library was prepared using innovative library preparation process in which PCR reactions were performed in real-time PCR machines to control cycles and limit PCR chimera formation. The final PCR products were quantified with qPCR fluorescence readings and pooled together based on equal molarity. The final pooled library was cleaned up with the Select-a-Size DNA Clean & Concentrator (Zymo Research, Irvine, CA, USA), then quantified with TapeStation (Agilent Technologies, Santa Clara, CA, USA) and Qubit (Thermo Fisher Scientific, Waltham, WA, USA). The final library was sequenced on Illumina MiSeq with a v3 reagent kit (600 cycles). Calculations of alpha and beta diversity were performed by standard methodology by Zymo Research.

Statistical Analysis. One-way ANOVA, with Bonferroni’s multiple comparisons test, was used for analysis of data. Wound healing assay data were analyzed using a Kruskal–Wallis test with Dunn’s multiple comparisons. Two-way ANOVA with Bonferroni’s multiple comparisons was used for analysis of proliferation data. Differences were considered statistically significant for *p*-values < 0.05. Because experiments were conducted over a period of up to 7 days, we do not expect there to have been secondary effects and outcomes on microbiome composition. All data points are included, and no outlying data were omitted. Power analysis, where we calculated the minimum sample size needed to detect statistical significance, was based on pilot experiments conducted by our research group. All analyses were performed using GraphPad Prism v6.0.4 software (GraphPad, San Diego, CA, USA). Data are presented as the mean ± standard error of the mean (SEM).

## 3. Results

### 3.1. A Mouse Model of Oro-Nasal Fistula (ONF)

**Figure 1:**
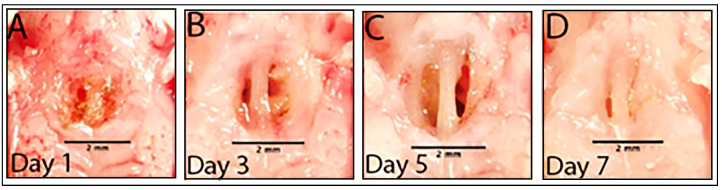
Mouse model of ONF demonstrating the occurrence of a fistula. (**A**,**B**) Day 1 and day 3 after ONF wound infliction. (**C**,**D**) Gradual narrowing of the fistula at 5 days and final ONF formation at day 7. Scale bar = 2 mm.

To develop a mouse model of an oro-nasal fistula (ONF), 10-week-old C57BL/6 mice were purchased from Jackson Laboratories and acclimatized at the Emory University mouse vivarium for two weeks. The 12-week old C57BL/6 mice were inflicted with a midline 1.5 mm injury in the hard palate. The initial injury was associated with exposure of the midline vomer bone, like that in humans, which narrowed over 7 days, as is the case in human healing (Figure 1A,B). Analysis of the hard palate through the ONF at day 5 revealed detectable tissue re-epithelialization at the edges of the wound (Figure 1C,D). However, healing did not completely seal the wound, and an opening between the oral cavity and the nasal cavity persisted, as is the case in the formation of an ONF in humans. This approach establishes a faithful model for human ONF in mice.

### 3.2. Oral Microbiome Changes During ONF Wound Healing

**Figure 2:**
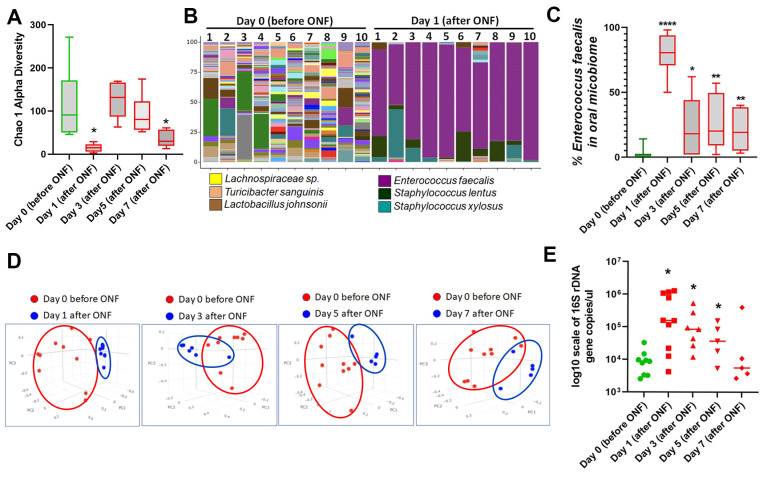
Analysis of the microbiome composition within the oral cavity of ONF wound model mice. (**A**) Measurement of the alpha diversity within oral microbiome samples before (day 0) and after ONF wound infliction for 7 days. Note that the Chao1 alpha diversity measures the microbial diversity of each sample and is a representation of the number of observed species in the samples. (**B**) Histogram representation of the alpha diversity within oral microbiome samples before (day 0) and at day 1 after ONF wound infliction. Note expansion in *Enterococcus faecalis* at day 1 after ONF formation. (**C**) Percentage of the total microbiome that is attributed to *Enterococcus faecalis* within the oral microbiome before (day 0) and for up to 7 days after ONF wound infliction. (**D**) Change in microbiome beta diversity in the oral cavity of mice subjected to an ONF for up to 7 days, compared to microbiome diversity at day 0 before wound infliction. Beta diversity is a measurement of microbial diversity differences between samples. The figure is a three-dimensional Principal Coordinate Analysis (PCoA) plot created using the matrix of paired-wise distance between samples calculated by the Bray–Curtis dissimilarity using unique amplicon sequence variants (ASV). Note: ONF results in a persistent change in the microbiome composition over at least 7 days. (**E**) The absolute abundance of bacterial (16S) DNA measured in the oral cavity at day 0 before ONF and after ONF wound infliction for 7 days. Note that ONF resulted in increased total bacterial burden in the oral cavity with significantly higher 16S rDNA copy numbers per microliter detected after ONF. For mice in (**A**–**E**), day 0 (*n* = 10), day 1 (*n* = 8), day 3 (*n* = 6), day 5 and 7 (*n* = 5). Statistical analysis by *t*-test. * *p* ≤ 0.05, ** *p* ≤ 0.01, **** *p* ≤ 0.0001.

Our research group previously reported that the microbiome composition changes following a biopsy wound to colonic tissue [8]. Based on this premise, we characterized the oral microbiome composition in mice before and after ONF injury. We show first that creation of an ONF in mice resulted in significant lowering of oral microbiome alpha diversity, with concurrent blooms of *Enterococcus faecalis*, *Staphylococcus lentus*, and Staphylococcus xylosus in the oral cavity (Figure 2A–C). We also show by beta diversity analysis that an ONF resulted in a persistent change in the microbiome composition up to 7 days post-injury (Figure 2D). Furthermore, an ONF resulted in increased total bacterial burden in the oral cavity with a significantly higher 16S rDNA copy number per microliter sample detected after ONF (Figure 2E). These data demonstrate the occurrence of a significant change in the oral microbiome after ONF formation.

### 3.3. Antibiotic Treatment Impacts the Oral Microbiome During ONF Wound Healing

**Figure 3:**
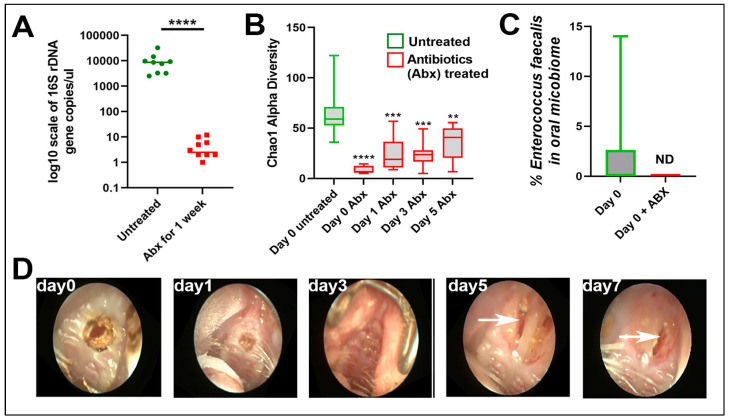
Antibiotic treatment impacts the oral microbiome during ONF wound healing. (**A**) The absolute abundance of bacterial (16S) DNA measured in the oral cavity mice treated with of antibiotics (Ampicillin, Vancomycin, Neomycin, Metronidazole) for 7 days, compared to untreated mice. (**B**) Chao1 alpha diversity within the oral microbiome of mice treated with antibiotic as described in (**A**) and subjected to an ONF as described in Figure 1. (**C**) Percentage of the total microbiome attributed to *Enterococcus faecalis* within the oral microbiome of mice treated with antibiotic as described in (**A**) at day 0 before ONF, compared to untreated controls. ND = not detected. (**D**) Wound healing of ONF in mice treated with antibiotics for up to 7 days post-wound infliction. Note: ONF is still visible (white arrows) in mice treated with antibiotics after 7 days. Images are a representative of 10 biological replicates. Statistical analysis by *t*-test. *n* = 10, ** *p* ≤ 0.01, *** *p* ≤ 0.001, **** *p* ≤ 0.0001.

*E. faecalis* is associated with *oral* diseases, such as caries, endodontic infections and periodontitis [9,10]. However, the implications of changes to the oral microbiome composition, including loss of taxa diversity or alpha diversity, or the bloom of other taxa such as *E. faecalis*, for tissue healing processes remain unstudied. Moreover, whether interactions of specific oral microbes with host cells activate pro-restitutive cellular signaling pathways in the oral wound bed is also unknown. To this end, we treated mice with a cocktail of 1 mg/mL Ampicillin, 0.5 mg/mL Vancomycin, 1 mg/mL Neomycin, and 1 mg/mL Metronidazole included in their drinking water, which are absorbable and non-absorbable antibiotics commonly used in experimental science to deplete the murine microbiome of normal viability [11], for 1 week and detected a >99.9% lowering in total bacterial burden (Figure 3A). We also detected an expected reduction in the microbiome alpha diversity (Figure 3B). Antibiotic pre-treatment rendered *E. faecalis* undetectable in the oral microbiome at day 0 (before ONF), and throughout the 7 days over which the rate of ONF healing was followed (Figure 3C). However, the antibiotic-mediated elimination of the bacterial load in the oral cavity did not impact ONF healing, with ONF remaining visible in the palate of mice at day 7 after wound infliction and antibiotic treatment (Figure 3D). These data suggest that antibiotics impact oral microbiome composition, but do not promote wound healing. The data collectively suggest that a dysbiotic microbiome, or loss of microbiome diversity, may both impair wound healing and contribute to the formation of an ONF.

## 4. Discussion

An ONF is a serious impediment to a child’s ability to eat and talk. Therefore, there is an urgent need to understand the microbiological context in which ONF healing occurs and how the oral microbiome can impact ONF healing. We report that a freshly inflicted wound in the oral palate of a mouse that models an open and unhealed ONF resulted in significant changes to the composition of the oral microbiome alpha diversity, including the expansion of reported oral pathogens. Treatment of mice with antibiotics prior to ONF infliction lowered microbiome alpha diversity and prevented microbial blooms, but did not improve ONF healing.

It is well established that wound healing on the skin and in the intestine is sensitive to local environmental factors, including the microbiome, where certain microbial community structures or pathogens can impede wound healing process [12]. Pathogens may inhibit wound healing by provoking a prolonged and unchecked inflammatory response. Mechanisms whereby this may occur include the consumption of vital nutrients needed for tissue repair, the direct damage and induced programed death of cells involved in healing, the secretion of bacterial factors that inhibit epithelial cell growth and cell migration, and the formation of biofilms that effectively shield pathogens from detection and elimination by immune cells [13]. All of these processes are likely mutually compatible in an environment where pathogens inhibit wound healing. Conversely, some bacteria have been reported to facilitate wound healing through mechanisms that include immune cell activation, the production of antimicrobial molecules, or the rebalancing of host healing factors such as MMPs. In this context, elimination of pro-restitutive bacteria extant within the microbiome by antibiotics may have an equally negative impact on oral wound healing as pathogens [8,14].

Overall, we contribute new knowledge about how a dysbiotic oral microbiome affects oral wound healing and showed that opportunistic pathogens quickly colonize the oral wound in mice. We also determined that antibiotics given prophylactically during surgery to eliminate pathogens lowers the microbial burden in the oral cavity but does not enhance wound healing of an ONF. Limitations to the current study include the use of mice as a model, since mice eat a single type of chow, and their oral microbiome is not subjected to nutritional variations typical of a human diet. In addition, although extensive measures were carried out for intraoperative and postoperative cleaning and care, including keeping the surgical site isolated with sterile drapes, mice unavoidably self-groom, which facilitates the introduction of environmental microbes to the oral cavity. Furthermore, a current limitation is that a conclusive mechanistic account explaining how both a bloom of pathogenic bacteria and the elimination of nearly all oral bacteria with antibiotics have similarly adverse effects on oral wound healing remains elusive.

## 5. Conclusions

This study yields new data to inform new considerations for the strategic use of peri-operative antibiotics in oral surgery and may be expanded to other clinical use cases related to oral wound healing following trauma and cancer surgery.

## Data Availability

The data used in this study are available upon request from the corresponding author.
